# Augmented PFKFB3-mediated glycolysis by interferon-γ promotes inflammatory M1 polarization through the JAK2/STAT1 pathway in local vascular inflammation in Takayasu arteritis

**DOI:** 10.1186/s13075-022-02960-1

**Published:** 2022-12-12

**Authors:** Rongyi Chen, Jinghua Wang, Xiaojuan Dai, Sifan Wu, Qingrong Huang, Lindi Jiang, Xiufang Kong

**Affiliations:** 1 Department of Rheumatology, Zhongshan Hospital Fudan University, Shanghai, 200032 China; 2 Center of Evidence-Based Medicine, Fudan University, No.180, Fenglin Road, Xuhui District, Shanghai, 200032 China

**Keywords:** Takayasu arteritis, Glucose metabolism, Macrophage, Inflammation, Interferon-γ

## Abstract

**Background:**

Takayasu arteritis (TAK) is characterized by pro-inflammatory M1 macrophage infiltration and increased interferon (IFN)-γ expression in vascular lesions. IFN-γ is a key cytokine involved in M1 polarization. Macrophage polarization is accompanied by metabolic changes. However, the metabolic regulation mechanism of IFN-γ in M1 macrophage polarization in TAK remains unclear.

**Methods:**

Immunohistochemistry and immunofluorescence were employed to observe the expression of IFN-γ, PFKFB3 (6-phosphofructo-2-kinase/fructose-2,6-biphosphatase 3, the rate-limiting enzyme in glycolysis), and macrophage surface markers in the vascular tissue. Monocyte-derived macrophages from patients with TAK were cultured to examine the role of PFKFB3 in IFN-γ-induced M1 macrophage polarization. Seahorse analysis was used to detect the alterations in glucose metabolism during this process. Quantitative reverse transcription PCR, flow cytometry, and western blot were used to confirm the phenotypes of macrophages and related signaling pathways.

**Results:**

In the vascular adventitia of patients with TAK, an increase in PFKFB3 accompanied by IFN-γ expression was observed in M1 macrophages. In vitro, IFN-γ successfully induced macrophage differentiation into the M1 phenotype, which was manifested as an increase in CD80 and HLA-DR markers and the pro-inflammatory cytokines IL-6 and TNF-α. During this process, PFKFB3 expression and glycolysis levels were significantly increased. However, glycolysis and M1 polarization induced by IFN-γ were suppressed by a PFKFB3 inhibitor. In addition, JAK2/STAT1 phosphorylation was also enhanced in macrophages stimulated by IFN-γ. The effects of IFN-γ on macrophages, including the expression of PFKFB3, glycolysis, and M1 polarization, were also inhibited by the JAK inhibitor tofacitinib or STAT1 inhibitor fludarabine.

**Conclusion:**

PFKFB3-mediated glycolysis promotes IFN-γ-induced M1 polarization through the JAK2/STAT1 signaling pathway, indicating that PFKFB3 plays an important role in M1 polarization mediated by IFN-γ; thus, PFKFB3 is a potential intervention target in TAK.

**Supplementary information:**

The online version contains supplementary material available at 10.1186/s13075-022-02960-1.

## Background

Takayasu arteritis (TAK) is a chronic, non-specific, granulomatous vasculitis characterized by inflammation of the aorta and its main branches, with an incidence of 0.4–3.4 per million around the world [1, 2]. The TAK treatment aims to control vascular inflammation and related pathological changes. According to current reports, the remission rates for conventional immunosuppressants and biologics in TAK were about 58% and 64% respectively. However, at least 50% patients suffered relapse and vascular complications within 10 years from the diagnosis [3, 4], indicating insidious progression of vascular lesions is still common in patients with TAK [5]. Therefore, it is necessary to explore the mechanism of vascular inflammation in TAK lesions and search for effective treatment targets.

TAK vascular lesions are infiltrated by various cells, most frequently macrophages, followed by CD4^+^ T cells, CD8^+^ T cells, and neutrophils [6]. Previously, we found that the macrophages in the active lesions of the vascular adventitia in TAK were predominantly of the M1 phenotype, and elevated CCL2 was also observed [7]. Simultaneously, various inflammatory cytokines including interferon (IFN)-γ and interleukin (IL)-6 were also increased in the vascular lesions [8]. IFN-γ is not only an activation marker of inflammatory Th1 cells in TAK [9] but also an important cytokine driving macrophage polarization [10, 11]. It is well known that IFN-γ induces macrophage differentiation into the M1 phenotype via the JAK/STAT1 signaling pathway and is involved in the pathogenesis of chronic inflammation and autoimmune diseases [12]. Recently, studies have shown that metabolic abnormalities play an important role in inflammation and disease development [13, 14]. However, the role of IFN-γ metabolism in M1 macrophage polarization remains unclear.

Glucose metabolism, which comprises glycolysis and oxidative phosphorylation, is pivotal in regulating macrophage polarization and the corresponding functions [15]. 6-Phosphofructo-2-kinase/fructose-2,6-biphosphatase 3 (PFKFB3) is a rate-limiting enzyme in glycolytic flux [16]. According to our preliminary RNA sequencing data, patients with TAK showed significantly increased PFKFB3 in peripheral blood mononuclear cells (data not published). It has been reported that PFKFB3 is increased in lipopolysaccharide (LPS)-stimulated macrophages, which may play a role in the pro-inflammatory function of M1 macrophages [17]. Whether PFKFB3-mediated glycolysis is also involved in the IFN-γ-induced macrophage polarization and whether this process contributes to the pro-inflammatory function of macrophages in TAK remain elusive.

Therefore, the present study investigates the glucose metabolism profiles and the central regulatory mechanisms of M1 macrophage polarization mediated by IFN-γ. The findings are expected to provide a basis for identifying an effective target to control vascular inflammation in TAK.

## Methods

### Patients and materials

This study included patients with TAK from Zhongshan Hospital affiliated to Fudan University. Patients with TAK were classified according to the 1990 American College of Rheumatology classification criteria [18]. Aortic specimens from 12 patients with TAK (4 patients in active status) and 9 control participants were used to study the protein expression in tissues, whereas peripheral blood from 37 patients with TAK (2 patients in active status) was used for further mechanism exploration (Supplementary Table 1). The TAK vascular specimens from patients with TAK were obtained from vascular operations, whereas the apparently normal vascular specimens were obtained from the remanent tissue of the donors for the operation of heart or liver transplantation.

### Pathological staining

To evaluate aortic inflammation, hematoxylin–eosin staining was performed according to the manufacturer’s instructions (Servicebio, Wuhan, China) [19]. In addition, immunohistochemistry (IHC) was employed to assess the expression of IL-4, IFN-γ, and PFKFB3 using previously described methods [19] and antibodies specific to IL-4 (Abcam, Cambridge, UK), IFN-γ (Abcam), and PFKFB3 (Abcam). Furthermore, multicolor immunofluorescence techniques were used to detect the co-expression of IFN-γ and PFKFB3 in CD68^+^ macrophages by Servicebio Technology (Wuhan, China) [20], using rabbit anti-human IFN-γ (Proteintech, Rosemont, IL), rabbit anti-human PFKFB3 (Abcam), and mouse anti-human CD68 (Abcam). The slides were scanned by a Pannoramic MIDI scanner (3DHISTECH, Hungary) and viewed using CaseViewer software v2.2. The positive areas in IHC images and mean fluorescence intensity (MFI) from immunofluorescence images were determined by three independent investigators (Rongyi Chen, Jinghua Wang, and Sifan Wu) using ImageJ software, for quantitative analysis.

### Cell isolation, culture, and treatment

Peripheral blood mononuclear cells were obtained from peripheral whole blood using the Ficoll-Paque method (Cytiva, Marlborough, MA). Subsequently, the primary monocytes were enriched with anti-CD14 microbeads (Milltenyi Biotech, Sunnyvale, CA, USA) and seeded in plates at 5 × 10^5^ cells/ml. RPMI-1640 cell culture medium and 10% fetal bovine serum (Grand Island, NY, USA) as well as 1% penicillin–streptomycin (KeyGEN Biotech, Nanjing, China) supplemented with 40 ng/ml macrophage colony-stimulating factor (M-CSF) (Absin, Shanghai, China) was used as a differentiation medium. The culture medium was changed on the third day, and after 5 days, adherent macrophages were obtained. Then, M0 macrophages were induced into M1 macrophages in a culture medium supplemented with 50 ng/ml LPS (Millipore Sigma, Burlington, MA, USA) and 20 ng/ml IFN-γ (Novus, Centennial, CO, USA) or M2 macrophages in a culture medium supplemented with 20 ng/ml IL-4 (Novus) and 20 ng/ml IL-13 (Novus) for 48 h [21]. In M1 polarization (induced by IFN-γ and LPS) experiments, to explore the role of IFN-γ in this process, LPS (50 ng/ml) was added in all the cell culture as a basal culture ingredient. To examine the role of PFKFB3 in macrophage polarization, PFKFB3 inhibitor PFK-015 (Selleck, Houston, TX, USA) was used. The JAK inhibitors tofacitinib (10 nM, Selleck) and baricitinib (50 nM, Selleck) and STAT1 inhibitor fludarabine (1 nM, Selleck) were also employed to validate the signaling pathway.

### Quantitative reverse transcription PCR

Total RNA was extracted from the cell pellet using TRIzol reagent (Invitrogen, Carlsbad, CA, USA) and reverse-transcribed into cDNA using the PrimeScript RT Master Mix (Takara, Shiga, Japan) according to the manufacturer’s protocol. Quantitative PCR was performed on the Applied Biosystems QuantStudio 5 Real-Time PCR System (Thermo Fisher Scientific, IL, USA) using SYBR Green reagents (Yeasen Biotech, Shanghai, China); the corresponding primers are listed in Supplementary Table 2. The expression of target genes was calculated using the 2^−ΔΔt^ method after normalizing to the housekeeping gene β-actin and control group.

### Flow cytometry

Macrophages were collected by Accutase cell detachment solution (STEMCEll Technology, Vancouver, Canada). M1 and M2 phenotypes were detected by flow cytometry. In this process, cells were stained with fluorescence-conjugated monoclonal antibodies including CD14-PE-Cy7, CD68-PE, CD80-BV510, HLA-DR-Percp/Cy5.5, CD163-BV421, and CD206-APC, together with LIVE/DEAD Fixable Dead Cell APC/Cy7 (all from Thermo Fisher Scientific) according to the manufacturer’s protocol. For the expression of PFKFB3 in different macrophage subtypes, the primary rabbit anti-human PFKFB3 and goat anti-rabbit secondary antibody conjugated with Alexa Fluor 488 (Jackson Laboratory, Bar Harbor, ME, USA) were employed to perform the intracellular staining with the fixation/permeabilization solution (BD Bioscience) according to the protocol of manufacture. The fluorescence was detected by the BD LSRFortessa flow cytometer (BD Bioscience), and the data were analyzed by FlowJo X (BD Bioscience).

### Glucose metabolism detection

To detect the glucose metabolism profiles of macrophages, the extracellular acidification rate (ECAR) and oxygen consumption rate (OCR) were examined using the glycolysis stress kit and Mito stress kit with the Seahorse XF96 Extracellular Flux Analyzer according to the manufacturer’s protocol. The results were generated using Seahorse Wave Desktop software (Agilent Technologies, Santa Clara, CA, USA).

### Western blot

Western blot was performed as previously described [19]. In brief, the protein extracted from the macrophages was degenerated, electrophoresed, and transferred to the polyvinylidene fluoride membrane (Millipore). Subsequently, the membrane was blocked using 5% non-fat milk and incubated with primary antibodies—rabbit anti-PFKFB3 (Abcam), iNOS (inducible nitric oxide synthase) (Proteintech), CD206, Jak1, P-Jak1, Jak2, P-Jak2, Jak3, P-Jak3, Stat 1, P-stat1 (Y701), P-stat1 (Y727) (all from CST technology), and mouse anti-β-actin (Abcam)—overnight at 4 °C. On the following day, membranes were washed and incubated with the corresponding horseradish peroxidase-conjugated secondary antibodies (Jackson Laboratory) and detected with an ECL reagent (Yeasen, Shanghai, China) using the chemiluminescence system (Tanon, Shanghai, China). The grayscale of the detected bands was assessed by ImageJ software (National Institutes of Health), and the results were expressed as fold changes after normalizing to β-actin.

### Enzyme-linked immunosorbent assay and lactate detection

The supernatant of the cell culture medium was obtained to detect the level of IL-1β, IL-6, IL-10, and TNF-α using the corresponding ELISA kits (all from KND Biotech, Quanzhou, Fujian, China). To detect the degree of glycolysis, the levels of lactate—the product of glycolysis—in the cell culture supernatant were examined using the lactate assay kit (Nanjing Jiancheng Bioengineering Institute, Nanjing, China).

### Statistical analysis

The data are presented as mean ± standard error of mean (*SEM*). A *t*-test was used to compare the difference between the two groups, whereas a one-way ANOVA analysis of variance together with multiple post hoc comparisons was employed to compare the difference among two more groups. A Spearman correlation analysis was used to analyze the relationship between the two continuous variants that were non-normally distributed. GraphPad software was used to perform the statistical analysis and to generate the corresponding graphs. The data used for analysis were based on at least three repeated experiments. A *p* value < 0.05 with two-sided tests was considered to indicate statistical significance.

## Results

### Increased PFKFB3 accompanied by IFN-γ in macrophages of active vascular lesions in TAK

**Fig. 1:**
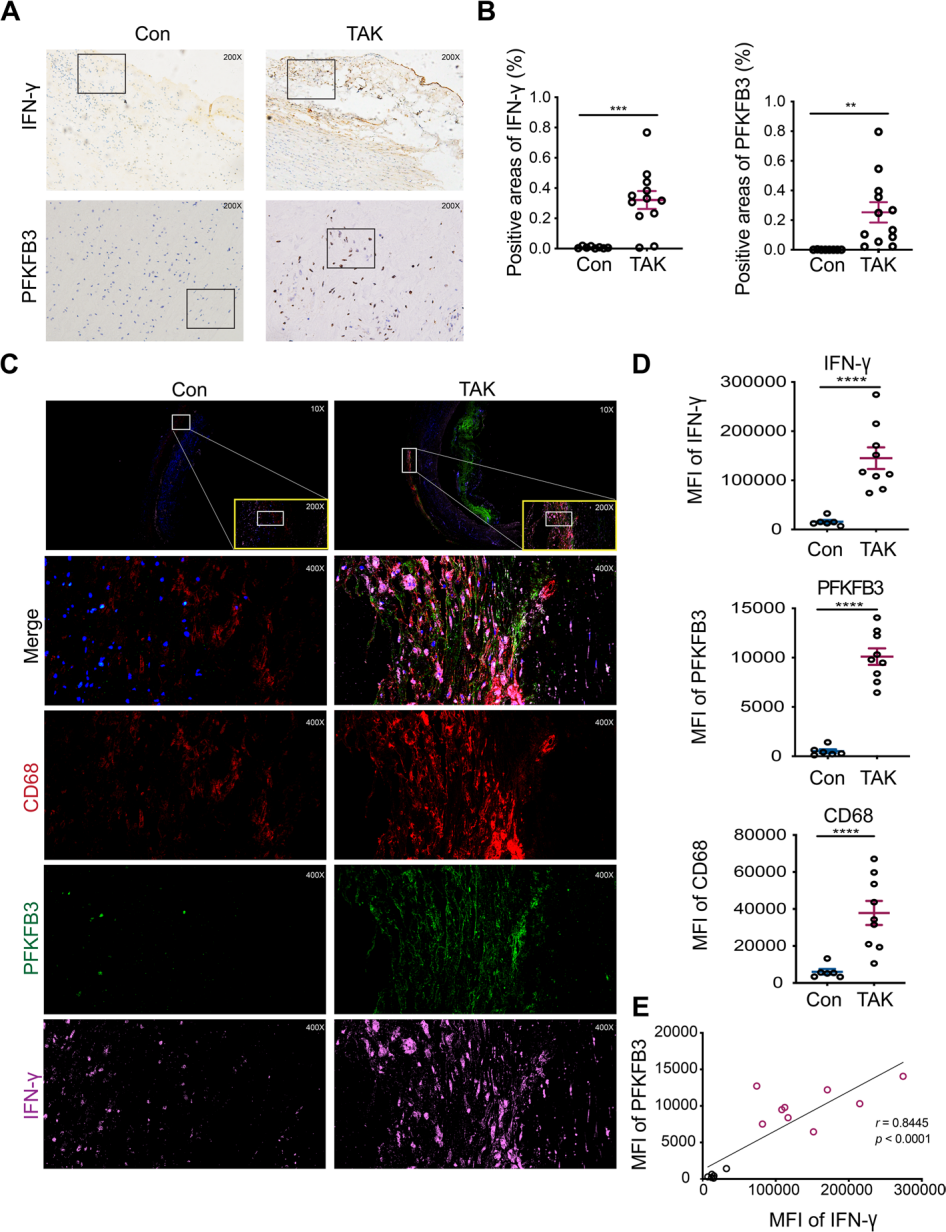
Expression and co-localization of 6-phosphofructo-2-kinase/fructose-2,6-biphosphatase 3 (PFKFB3) and interferon (IFN)-γ in macrophages of the adventitia in Takayasu arteritis (TAK). **A**, **B** Expression of IFN-γ and PFKFB3 in the aortic adventitia (*n* = 12 for TAK, *n* = 8 for control); **C**, **D** expression of IFN-γ, CD68, and PFKFB3 in TAK adventitia (*n* = 9 for TAK; *n* = 6 for control); **E** the Spearman correlation analysis of the expression (MFI) between IFN-γ and PFKFB3. MFI mean fluorescence intensity. Data revealed as *mean* ± *SEM*; *p < 0.05; **p < 0.01; ***p < 0.001; ****p < 0.0001

IHC staining showed that PFKFB3 and IFN-γ expressions were significantly increased in the adventitia as well as media in TAK vascular tissues (*p* < 0.05; Fig. 1A, B; Supplementary Figure 1A-B). To further investigate the relationship between IFN-γ and PFKFB3 in macrophages, multicolor immunofluorescence analysis was performed. The results indicated that the CD68^+^ macrophages were mainly infiltrated in the inflammatory zone of TAK adventitia, which was consistent with our previous study [7]. In addition, both PFKFB3 and IFN-γ were upregulated in CD68^+^ macrophages in TAK vascular lesions compared with the control (*p* < 0.0001; Fig. 1C, D; Supplementary Figure 2). Furthermore, the expression of PFKFB3 and IFN-γ was positively correlated in the adventitia of TAK vascular tissue (*r* = 0.8445, *p* < 0.0001, Fig. 1E).

### Selectively increased expression of PFKFB3 in M1 macrophages induced by IFN-γ in vitro

**Fig. 2:**
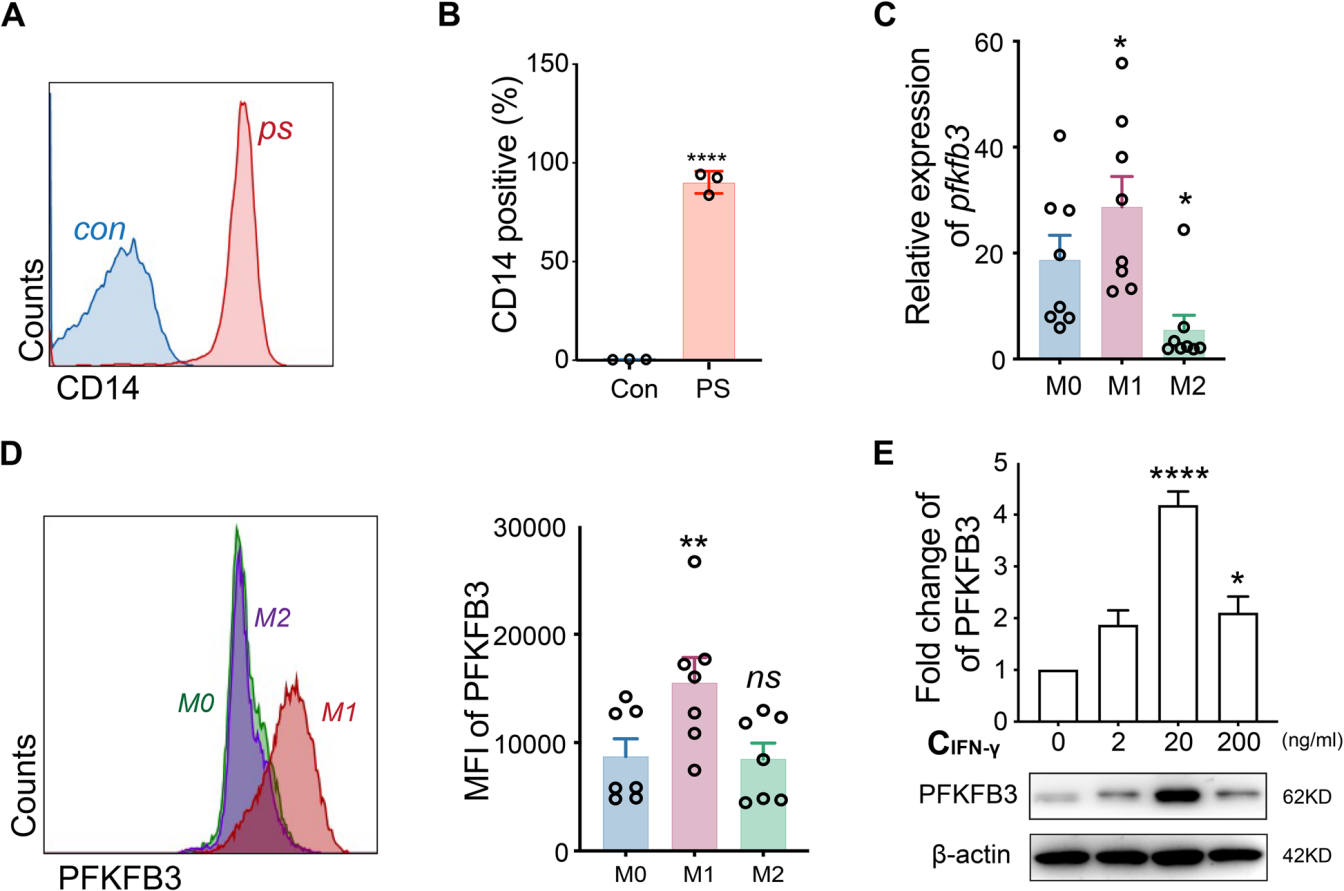
Relationship between interferon (IFN)-γ and 6-phosphofructo-2-kinase/fructose-2,6-biphosphatase 3 (PFKFB3) in M1 macrophages in vitro. **A**, **B** CD14.^+^ cell selection by flow cytometry (*n* = 3; con, control; ps, CD14-positive selected cells); **C**, **D** expression of PFKFB3 in M1 (induced by 20 ng/ml IFN-γ and 50 ng/ml LPS) and M2 (induced by 20 ng/ml IL-4 and 20 ng/ml IL-13) macrophage subtypes by quantitative reverse transcription PCR and flow cytometry; **E** effect of different concentrations of IFN-γ (0, 2, 20, 200 ng/ml) with LPS (50 ng/ml) on the expression of PFKFB3 in M1 differentiation. Data revealed as *mean* ± *SEM*, *p < 0.05; **p < 0.01; ***p < 0.001; ****p < 0.0001

Because tissue macrophages are mainly derived from circulating monocytes, monocytes obtained from patients with TAK were used to perform in vitro studies (Fig. 2A, B). M0 macrophages treated with LPS (50 ng/ml) and IFN-γ (20 ng/ml) showed M1 polarization, with an increased expression of HLA-DR and CD80 and pro-inflammatory markers IL-6 and TNF-α (Supplementary Figure 3A–E, 4A–C, 4E, F). During this process, PFKFB3 expression was significantly increased (Fig. 2C, D). In addition, PFKFB3 expression was also verified in M1 polarization with different IFN-γ concentrations accompanied with LPS. The results indicated that PFKFB3 expression was gradually increased with the increase of IFN-γ concentration, with the optimal concentration of 20 ng/ml (Fig. 2E). To verify the role of PFKFB3 in macrophage polarization, PFKFB3 levels in M2 macrophages were also detected. After inducing M0 macrophages with IL-4 (20 ng/ml) and IL-13 (20 ng/ml), the M2 phenotype with the CD163 and CD206 markers was obtained. However, no increase of PFKFB3 was observed in M2 macrophages, indicating that PFKFB3 was selectively expressed in M1 macrophages (Fig. 2C, D; Supplementary Figures 3 and 4D).

### Increased glycolysis mediated by PFKFB3 during M1 polarization induced by IFN-γ

**Fig. 3:**
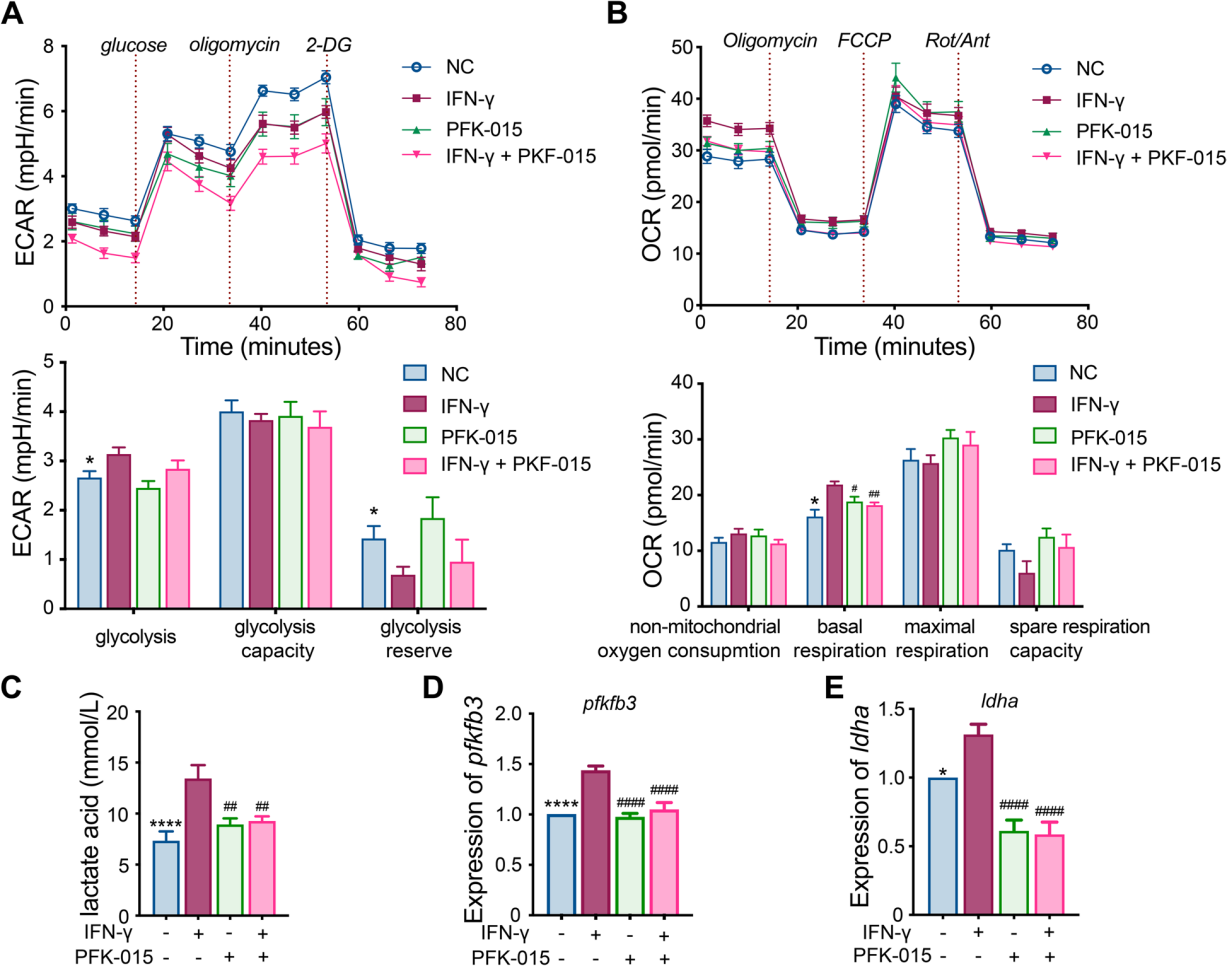
Glucose metabolism profile in M1 macrophage polarization in TAK. **A** Extracellular acidification rate (ECAR) was detected during M1 macrophage polarization and the inhibitory effect of PFK-015 (a 6-phosphofructo-2-kinase/fructose-2,6-biphosphatase 3 inhibitor, 10 μM) was examined in this process; **B** oxygen consumption rate (OCR) was detected in M1 macrophage polarization and the inhibitory effect of PFK-015 (10 μM) was examined in this process; **C** lactate levels were detected in the culture supernatant of M1 macrophages stimulated by IFN-γ and the inhibitory effect of PFK-015 (10 μM) in this process was evaluated; **D**, **E** gene expression of *PFKFB3* and *LDHA* was detected by RT-PCR in M1 macrophages and the inhibitory effect of PFK-015 (10 μM) was examined in this process. LPS (50 ng/ml) was added in all the groups besides the IFN-γ (20 ng/ml) or PFK-015 (10 μM) as indicated in the figures. Data revealed as *mean* ± *SEM*, *p < 0.05; **p < 0.01; ***p < 0.001; ****p < 0.0001; #*p* < 0.05; ##*p* < 0.01; ###*p* < 0.001; ####*p* < 0.0001

Because PFKFB3 is involved in glucose metabolism, the ECAR and OCR in polarized macrophages were investigated. The results suggested that glycolysis was significantly enhanced in macrophages treated with IFN-γ plus LPS (Fig. 3A, B). Consistent with this phenomenon, lactate was also significantly increased in macrophages stimulated with IFN-γ (Fig. 3C). Moreover, the expression of genes involved in the downstream and upstream pathways of glycolysis, including *PFKFB3*, *LDHA*, and *GLUT1*, was upregulated in M1 macrophages (Fig. 3D, E, Supplementary Figure 4G). However, these effects on glycolysis, lactate levels, and *LDHA* and *GLUT1* expression were significantly inhibited in the presence of PFKFB3 inhibitor PFK-015 (Fig. 3C–E, Supplementary Figure 4G). In addition, macrophage activity was also decreased in the presence of PFKFB3 inhibitor PFK-015 (Supplementary Figure 5A-B).

Analysis of OCR showed that IFN-γ elevated the basal respiration rate of macrophages, and the effect was inhibited by PFK-015. However, the glycolysis capacity and maximal respiration did not significantly change (Fig. 3A, B).

### M1 polarization induced by IFN-γ was suppressed by PFKFB3 inhibitor

**Fig. 4:**
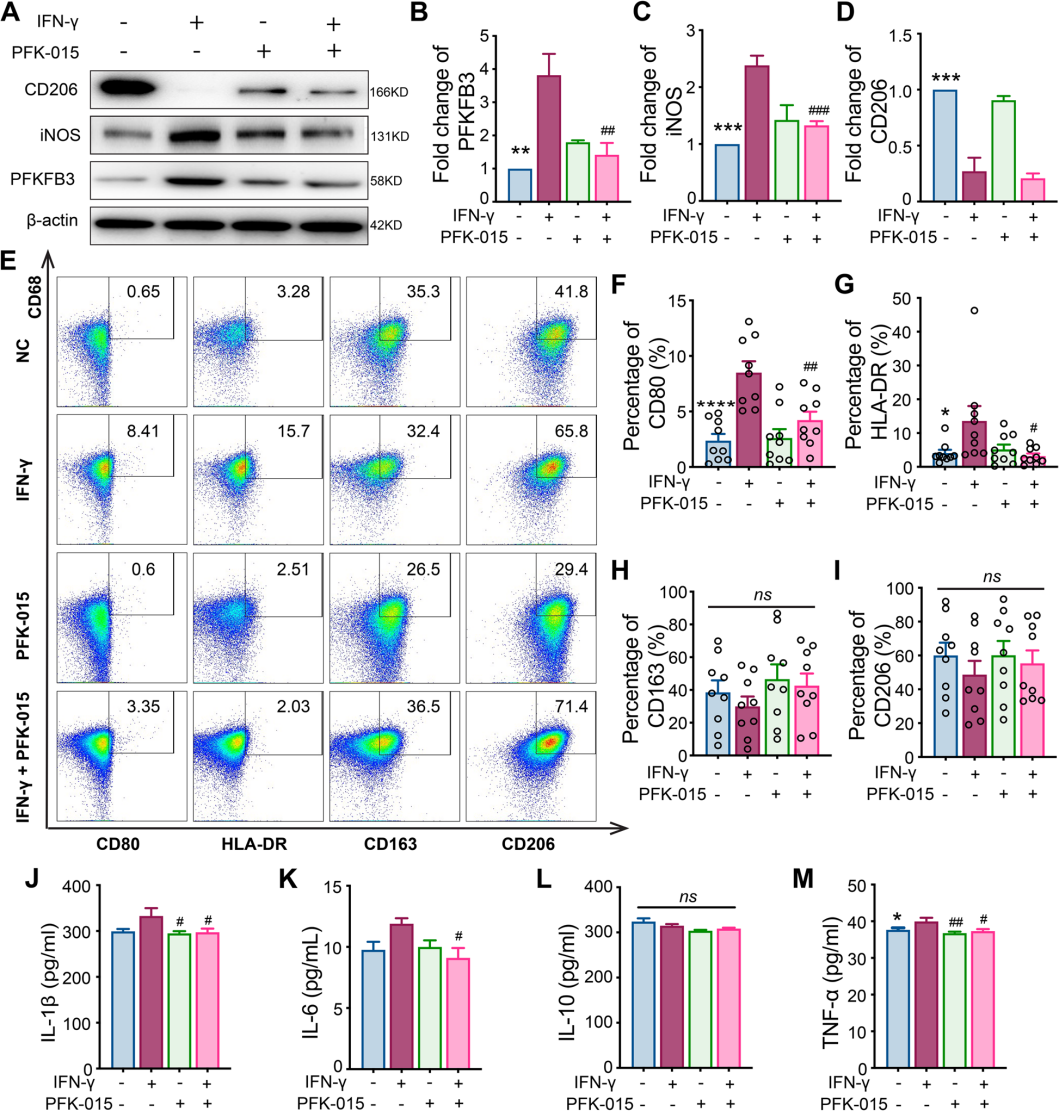
Effect of 6-phosphofructo-2-kinase/fructose-2,6-biphosphatase 3 (PFKFB3) inhibition on M1 macrophage polarization and inflammatory cytokine production. **A**–**D** Changes in the expression of PFKFB3, iNOS, and CD206 were detected in M1 polarization by western blot and the inhibitory effect of PFKFB3 inhibitor PFK-015 (10 μM) was examined in this process; **E**–**I** expression of CD80, HLA-DR, CD163, and CD206 were detected by flow cytometry in M1 macrophage polarization, and the role of PFKFB3 was evaluated by applying its inhibitor PFK-015 (10 μM) during this process; **J**–**M** levels of different cytokines (IL-1β, IL-6, IL-10, and TNF-α) were detected by ELISA in culture supernatant during M1 polarization and the role of PFKFB3 was evaluated by applying its inhibitor PFK-015 (10 μM) during this process; *n* = 9. LPS (50 ng/ml) was added in all the groups besides the IFN-γ (20 ng/ml) or PFK-015 (10 μM) as indicated in the figures. Data revealed as *mean* ± *SEM*, *p < 0.05; **p < 0.01; ***p < 0.001; ****p < 0.0001; #*p* < 0.05; ##*p* < 0.01; ###*p* < 0.001

Subsequently, the effect of PFKFB3 inhibition on macrophage phenotypes was investigated. Treatment with PFK-015 during IFN-γ-induced macrophage polarization reduced the expression of M1 surface markers CD80, iNOS, and HLA-DR (Fig. 4A–I; Supplementary Figure 4A–D, 6). Moreover, the inflammatory cytokines expressed by M1 macrophages, including IL-1β, IL-6, and TNF-α, were also suppressed by PFKFB3 inhibition (Fig. 4J–M, Supplementary Figure 4E-F).

### JAK/STAT1 signaling pathway is involved in the IFN-γ/PFKFB3-mediated M1 polarization

**Fig. 5:**
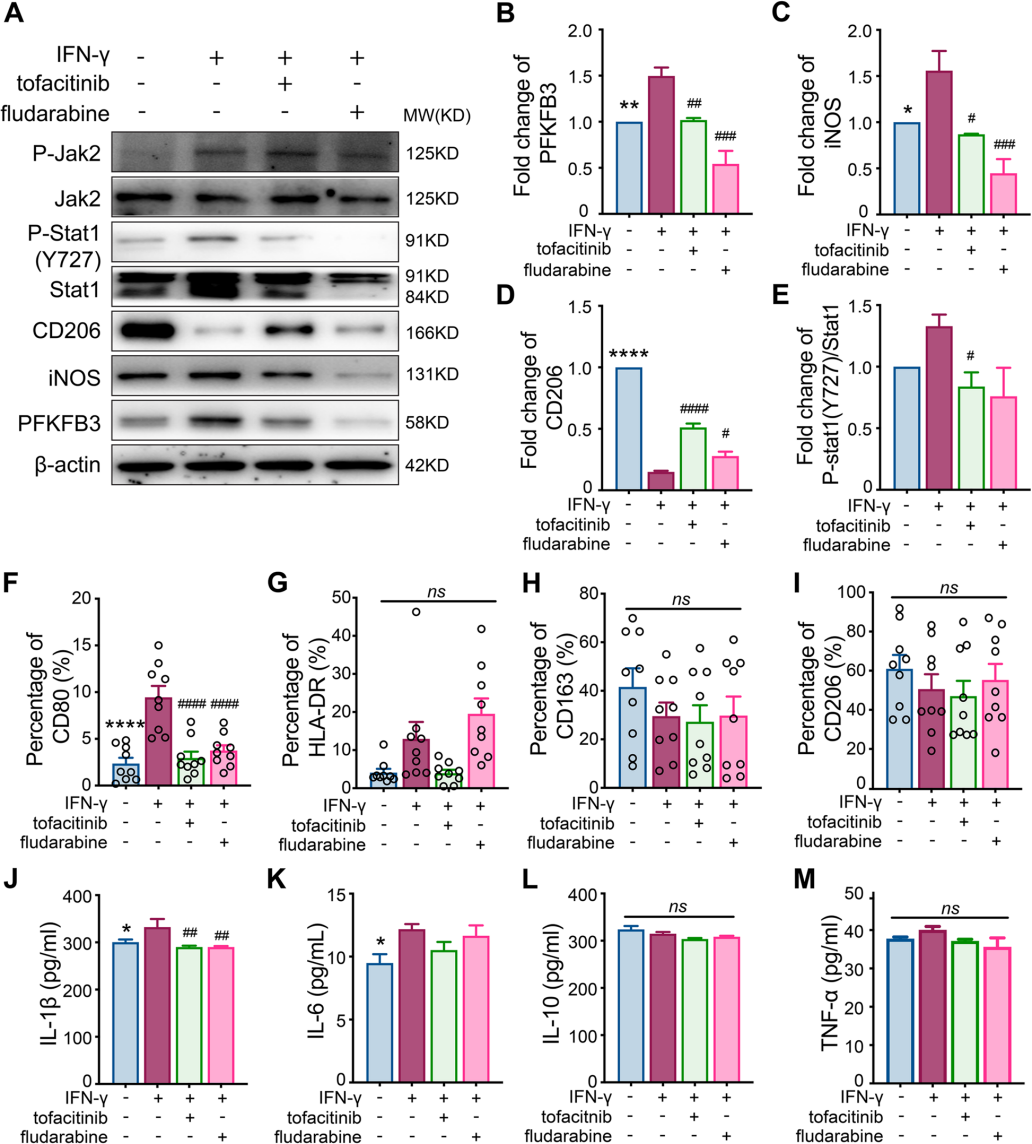
Signaling pathway of interferon (IFN)-γ mediating M1 polarization. **A**–**E** Change in PFKFB3, iNOS, CD206, and signaling pathway proteins during M1 macrophage polarization; **F**–**I** expression of CD80, HLA-DR, CD163, and CD206 were detected by flow cytometry in M1 macrophage in the presence of a JAK inhibitor (tofacitinib, 10 nM) and a STAT1 inhibitor (fludarabine, 1 nM); **J**–**M** different cytokines (IL-1β, IL-6, IL-10, and TNF-α) were detected in M1 macrophage culture supernatant in the presence of a JAK inhibitor (tofacitinib, 10 nM) and a STAT1 inhibitor (fludarabine, 1 nM); *n* = 8. LPS (50 ng/ml) was added in all the groups besides the IFN-γ (20 ng/ml) or tofacitinib (10 nM) or fludarabine (1 nM) as indicated in the figures. Data revealed as *mean* ± *SEM*, *p < 0.05; **p < 0.01; ***p < 0.001; ****p < 0.0001; ##*p* < 0.01; ###*p* < 0.001; ####*p* < 0.0001

Finally, the upstream signaling pathway regulating PFKFB3 was explored in IFN-γ-stimulated macrophages. We found that JAK2 and STAT1 (Y727) were significantly phosphorylated in macrophages stimulated by IFN-γ (Fig. 5A). When JAK was suppressed by tofacitinib, the phosphorylation of STAT1 was downregulated, the expression of PFKFB3 and basal glycolysis were inhibited, and lactate levels decreased as well (Fig. 5A–E, Supplementary Figures 7–8). Simultaneously, the levels of M1 macrophage markers iNOS and CD80 and inflammatory cytokines IL-1β and IL-6 declined significantly (Fig. 5F–I). Similarly, the M1 phenotype proteins CD80 and HLA-DR could also be inhibited by another Jak2 inhibitor baricitinib (Supplementary Figure 9, Supplementary Table 3). Moreover, similar basal glycolysis inhibition, M1 marker reduction, and decrease in inflammatory cytokines were observed when STAT1 was inhibited by fludarabine (Fig. 5J–M; Supplementary Figures 8, 10).

**Fig. 6:**
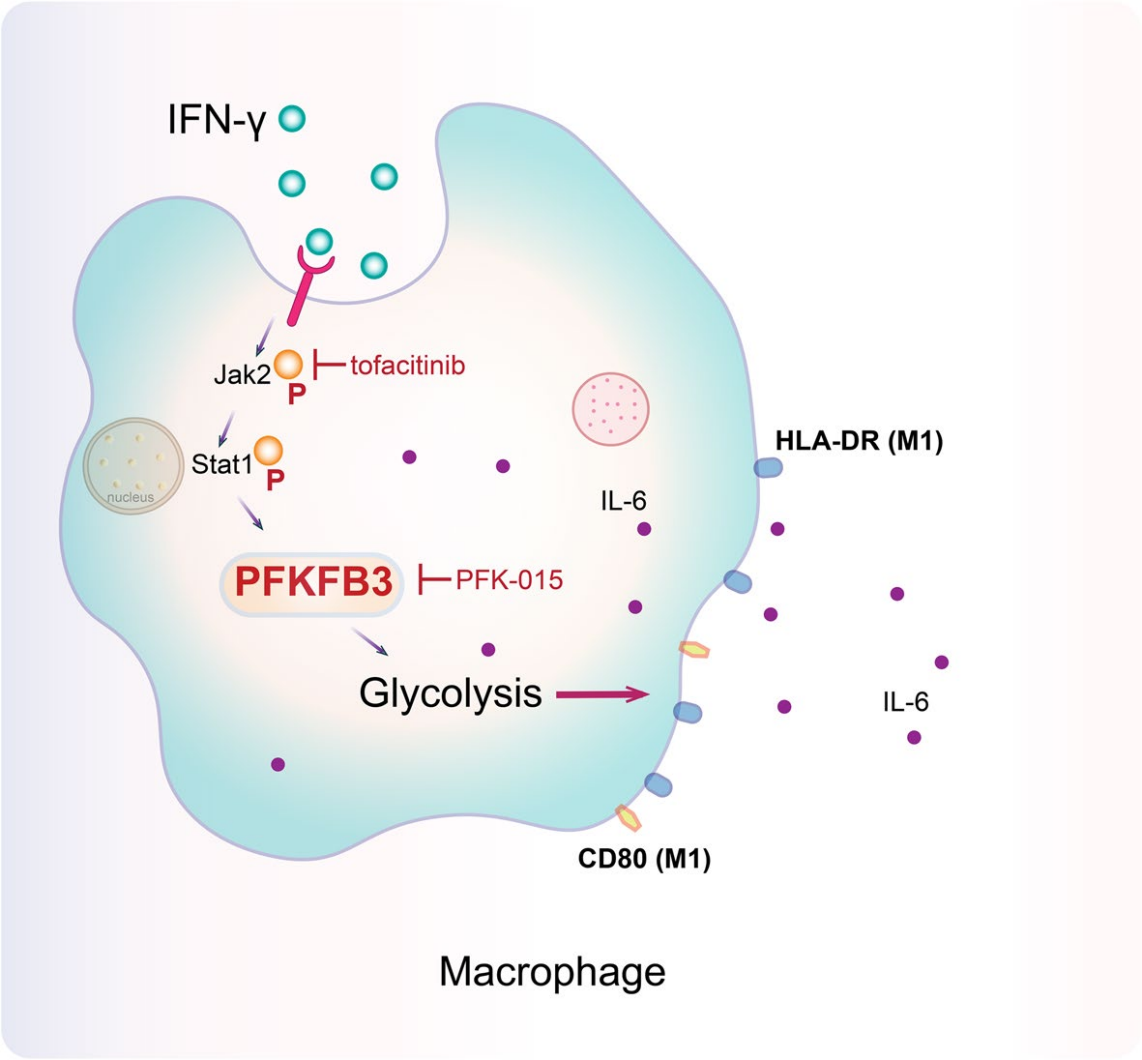
Schematic diagram showing that interferon (IFN)-γ promotes PFKFB3 expression and glycolysis, mediating M1 macrophage polarization and inflammation by JAK2/STAT1 phosphorylation, and this process could be inhibited by inhibitors tofacitinib (JAK2), fludarabine (STAT1), or PFK-015(PFKFB3)

A schematic diagram was illustrated to summarize the content of this study (Fig. 6).

## Discussion

This study found that IFN-γ could promote inflammatory M1 macrophage differentiation via enhancing PFKFB3-mediated glycolysis through JAK2/STAT1 signal pathway. This process might promote M1 macrophage-mediated inflammation in vascular adventitia lesions in TAK. As a whole, this study provides a novel mechanism for M1 polarization in TAK and sheds light on a new target for the treatment of TAK.

Cytokines and immune cells play a pivotal role in the regulation of inflammatory cascade responses in local immunity [22]. Previously, cytokines including IL-6 and TNF-α have been shown to drive pro-inflammatory M1 macrophage differentiation [23, 24], promoting the downstream inflammatory response. We previously demonstrated that IL-6 and IFN-γ levels increased with disease activity (Kerr score) in active TAK [25]. Ren et al. showed that IFN-γ, IL-6, IL-17, and TNF-α levels were significantly higher in patients with TAK than in healthy donors [8]. In the present study, IFN-γ levels and inflammatory M1 macrophages significantly increased in the inflammatory adventitia in TAK, which is consistent with the previous observations of serum levels and the dynamic transition of macrophages in TAK adventitia [7]. Therefore, IFN-γ and M1 macrophages likely play a pivotal role in the pathogenesis of TAK.

Macrophages are critical in the pathological process of TAK. Dos Santos et al. reported that fewer M1 macrophages than M2 macrophages were present in vascular walls [6]; however, we had observed that M1 macrophages increased in the adventitia of active untreated TA, whereas M2 macrophages increased in the media of treated TAK [7]. Similarly, in the present study, M1 macrophages were enriched in the adventitia. The different disease activity statuses, different macrophage phenotype surface markers, and limited sample size may account for this observation. However, these results suggest that M1 macrophages are structurally involved in the pathogenesis of TAK. Furthermore, augmented IFN-γ has been demonstrated to promote M1 macrophage development in vitro [26], which is consistent with the elevated IFN-γ and M1 macrophages in the adventitia of TAK. However, whether these IFN-γ-induced M1 macrophages were derived from circulating monocytes or tissue-resident naive macrophages remains unclear.

Macrophage polarization depends on energy metabolism. In TAK, PFKFB3 was highly expressed in polarized M1 macrophages and adventitial macrophages, accompanied by increased glycolysis under the stimulus of IFN-γ. The PFKFB3 inhibition reversed these effects and blocked the polarization of M1 macrophages. Thus, the results suggest that PFKFB3-mediated glycolysis is involved in IFN-γ-mediated M1 polarization, which leads to local vascular inflammation. However, enhanced mitochondrial activity is observed in IFN-γ-induced M1 macrophage differentiation. This effect is blocked by the PFKFB3 inhibitor PFK-015, which also reduces glucose uptake [27]. Artyomov et al. [28] reported the contradictory phenomenon of increased mitochondrial activity in peritoneal macrophages and reduced mitochondrial activity in bone marrow-derived macrophages, which may be attributed to the arginine metabolism tendency favoring iNOS and Arg-1 (arginase-1) in the two types of cells. Different metabolism profiles under different stimulus strategies and cell phenotypes may be the main reason for these different findings; however, further research is needed to illustrate the underlying mechanism. Thus, glycolysis increased in IFN-γ-mediated M1 polarization in TAK.

Enhanced glycolysis driven by PFKFB3 in macrophages is crucial in innate antiviral immune response, supporting the engulfment and removal of infected cells [29]. Moreover, enhanced glycolysis promotes M1 macrophage polarization to produce inflammatory cytokines such as IL-1β, IL-6, and TNF-α, which initiate and accelerate the pathogenesis of TAK [19, 30]. The PFKFB3 inhibitor PFK-015 was effective in blocking M1 macrophage polarization and decreasing the inflammatory cytokines such as IL-6 and TNF-α in M1 macrophages in TAK in the presence of IFN-γ. IL-6 is an important driving force in the progression of TAK because it activates aortic adventitial fibroblasts and promotes vascular fibrosis [19, 31]. Therefore, targeting M1 polarization via the IFN-γ/PFKFB3 pathway is significant for inflammation control and prognosis improvement in TAK treatment, which deserves more exploration and validation in future studies.

JAK/STAT is the predominant signaling pathway in the inflammatory response mediated by cytokines. JAK/STAT5 is involved in T cell activation in TAK [32], and STAT1 is phosphorylated in macrophages under the stimulus of IFN-γ [33, 34]. JAK2/STAT1 is activated when IFN-γ promotes M1 polarization, which is blocked by the JAK inhibitor tofacitinib and STAT1 inhibitor fludarabine. The blocking effect on M1 polarization markers of tofacitinib and baricitinib highlighted the significance of JAK inhibitors in TAK treatment [35]. But the inhibitory effect on glycolysis and glycolysis capacity was more prominent in tofacitinib treatment when comparing with those of fludarabine. This phenomenon indicates Jak was in the upstream of regulating glucose metabolism and might have other signaling pathways other than STAT1. Thus, inhibition of Jak was more effective than inhibition of STAT1. Moreover, tofacitinib has been demonstrated to antagonize the effect of IL-6 in promoting vascular fibrosis [19, 31] but also control inflammation and alleviate disease activity in patients with TAK [36, 37]. However, identifying the suitable patients for JAK inhibitors should be explored; moreover, it is important to identify novel, safe, and effective inhibitors targeting JAK/STAT.

## Conclusion

Increased PFKFB3 with glycolysis induced by IFN-γ promoted M1 polarization via the JAK2/STAT1 signaling pathway in TAK adventitia. Interventions targeting PFKFB3 or the upstream regulating signaling pathway were beneficial in controlling M1 polarization and inflammatory cytokine production, which may be conducive to TAK treatment in the future.

## Supplementary Information

**Additional file 1.**

## Data Availability

The datasets used and/or analyzed during the current study are available from the corresponding authors on reasonable request.

## Abbreviations

Arg-1: Arginase-1
ECAR: Extracellular acidification rate
IFN-g: Interferon-g
IHC: Immunohistochemistry
iNOS: Inducible nitric oxide synthase
OCR: Oxygen consumption rate
PFKFB3: 6-Phosphofructo-2-kinase/fructose-2,6-biphosphatase 3
TAK: Takayasu arteritis
M-CSF: Macrophage colony-stimulating factor
