# Supplementary material for: Augmented PFKFB3-mediated glycolysis by interferon-γ promotes inflammatory M1 polarization through the JAK2/STAT1 pathway in local vascular inflammation in Takayasu arteritis
Source: Arthritis Res Ther. 2022 Dec 12;24:266. doi: 10.1186/s13075-022-02960-1 (PMC9743547; doi:10.1186/s13075-022-02960-1)

***Supplementary Materials***

**Supplementary table 1.** Characteristics of the enrolled Takayasu arteritis patients.

| **Items** | ***in vitro*** | ***in vivo*** |
| --- | --- | --- |
| Patients | N= 37 | N=12 |
| Age, years | 32.4 ± 14.8 | 46 ± 15.3 |
| Gender (Female), n (%) | 32(86.5) | 6(50) |
| Naïve, n (%) | 2(5.4) | 8(66.7) |
| **Vascular types** |  |  |
| I | 7(18.9) |  |
| IIa | 3(8.1) | 6 |
| IIb | 2(5.4) | 1 |
| III | 4(10.8) |  |
| IV | 1(2.7) |  |
| V | 20(54.1) | 2 |
| **Disease activity** |  |  |
| Active status (NIH score >1) | 2(5.4) | 4(33.3) |
| ESR, mm/h | 5.0(2.0-13.5) | 30.1± 26.6 |
| CRP, mg/L | 1.1(0.5-5.0) | 9.4 ±10.1 |
| SAA, mg/L | 7.4(4.3-14.3) | / |
| C3, g/L | 1.03 ± 0.18 | / |
| C4, g/L | 0.22 ± 0.08 | / |
| CH50, g/L | 63.3 ± 14.3 | / |
| IL-6, pg/ml | 3.2(2.3-5.9) | / |
| TNF-α, pg/ml | 9.2(7.2-18.1) | / |
| **Interventions** |  |  |
| Prednisone, n (%) | 34(91.9) | 4(33.3) |
| Methotrexate, n (%) | 14(37.8) | / |
| Leflunomide, n (%) | 11(29.7) | / |
| Azathioprine, n (%) | 1(2.7) | / |
| Cyclophosphamide, n (%) | 1(2.7) | / |
| Thalidomide, n(%) | / | 3(25.0) |
| Tofacitinib, n (%) | 1(2.7) | / |
| Mycophenolate mofetil, n (%) | 1(2.7) | / |
| IL-6R mAb (tocilizumab), n (%) | 12(32.4) | 1(8.3) |
| TNF-α mAb (Suginmab, Adalimumab), n (%) | 5(13.5) | / |

Note: 1. The *in vivo* analysis consists of immunochemistry and immunofluorescence for vascular specimens.

2. The disease activity was judged by the Kerr score.

3. The intervention strategy indicates the drugs used within 3 months before biological samples obtained.

**Supplementary table 2.** The primers of related genes.

| ***Genes*** | **Forward primer** | **Reverse primer** |
| --- | --- | --- |
| *β-actin* | CATGTACGTTGCTATCCAGGC | CTCCTTAATGTCACGCACGAT |
| *pfkfb3* | GCCACAACTGTAGGGTCGT | GCCACAACTGTAGGGTCGT |
| *glut1* | GGCCAAGAGTGTGCTAAAGAA | ACAGCGTTGATGCCAGACAG |
| *ldha* | ATGGCAACTCTAAAGGATCAGC | CCAACCCCAACAACTGTAATCT |
| *inos* | TTCAGTATCACAACCTCAGCAAG | TGGACCTGCAAGTTAAAATCCC |
| *cd80* | AAACTCGCATCTACTGGCAAA | GGTTCTTGTACTCGGGCCATA |
| *hla-dr* | GAGCAGGTTAAACATGAGTGTCA | CTCTCCACAACCCCGTAGT |
| *arg-1* | GTGGAAACTTGCATGGACAAC | AATCCTGGCACATCGGGAATC |
| *Il-6* | ACTCACCTCTTCAGAACGAATTG | CCATCTTTGGAAGGTTCAGGTTG |
| *tnf-α* | CCTCTCTCTAATCAGCCCTCTG | GAGGACCTGGGAGTAGATGAG |

**Supplementary Figure 1.** The expression of IFN-γ and PFKFB3 in the media of the artery. (A) The expression of IFN-γ in the media; (B) The expression of PFKFB3 in the media. Con, control; TAK, takayasu arteritis; *p <0.05; **p<0.01.


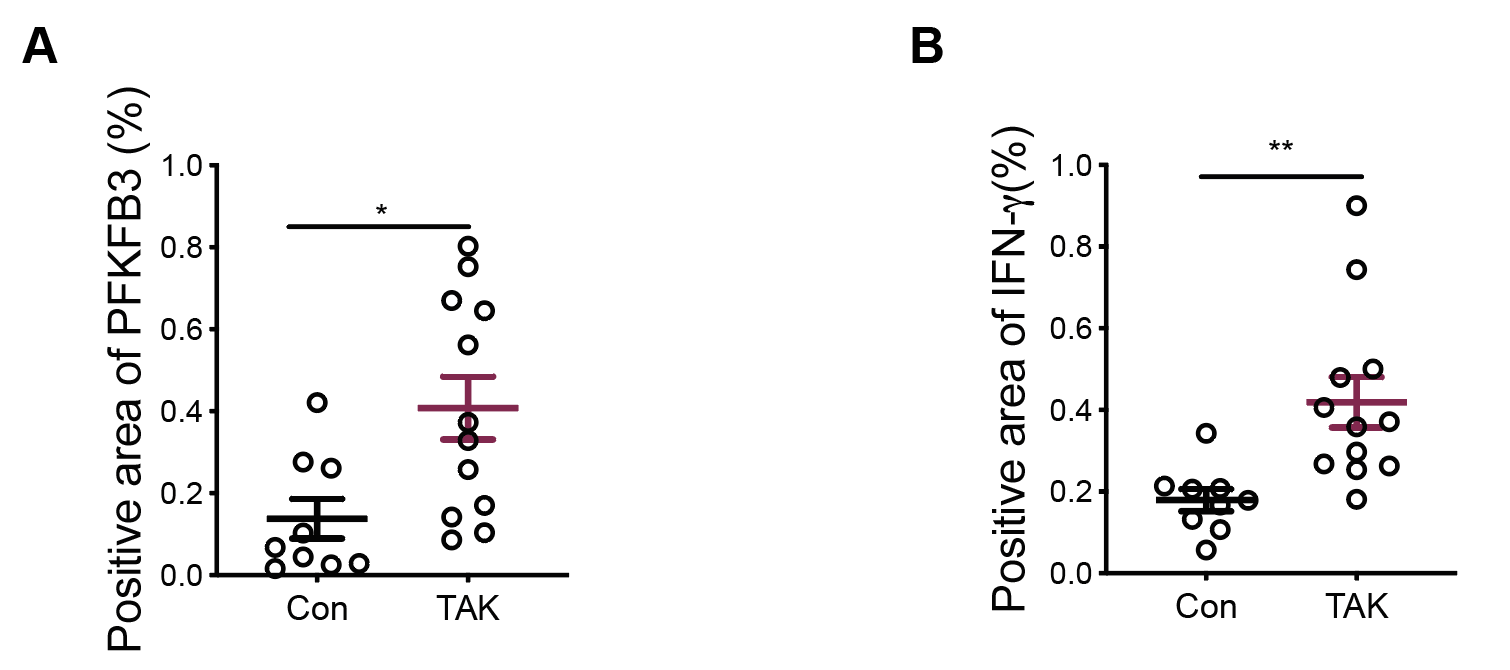


**Supplementary Figure 2.** Expression of PFKFB3 and col-localization of IFN-γ in TAK adventitia.

Magnified picture of **Figure 1C**, the col-localization of IFN-γ, CD68, and PFKFB3 in the aortic adventitia of TAK, ×2000;


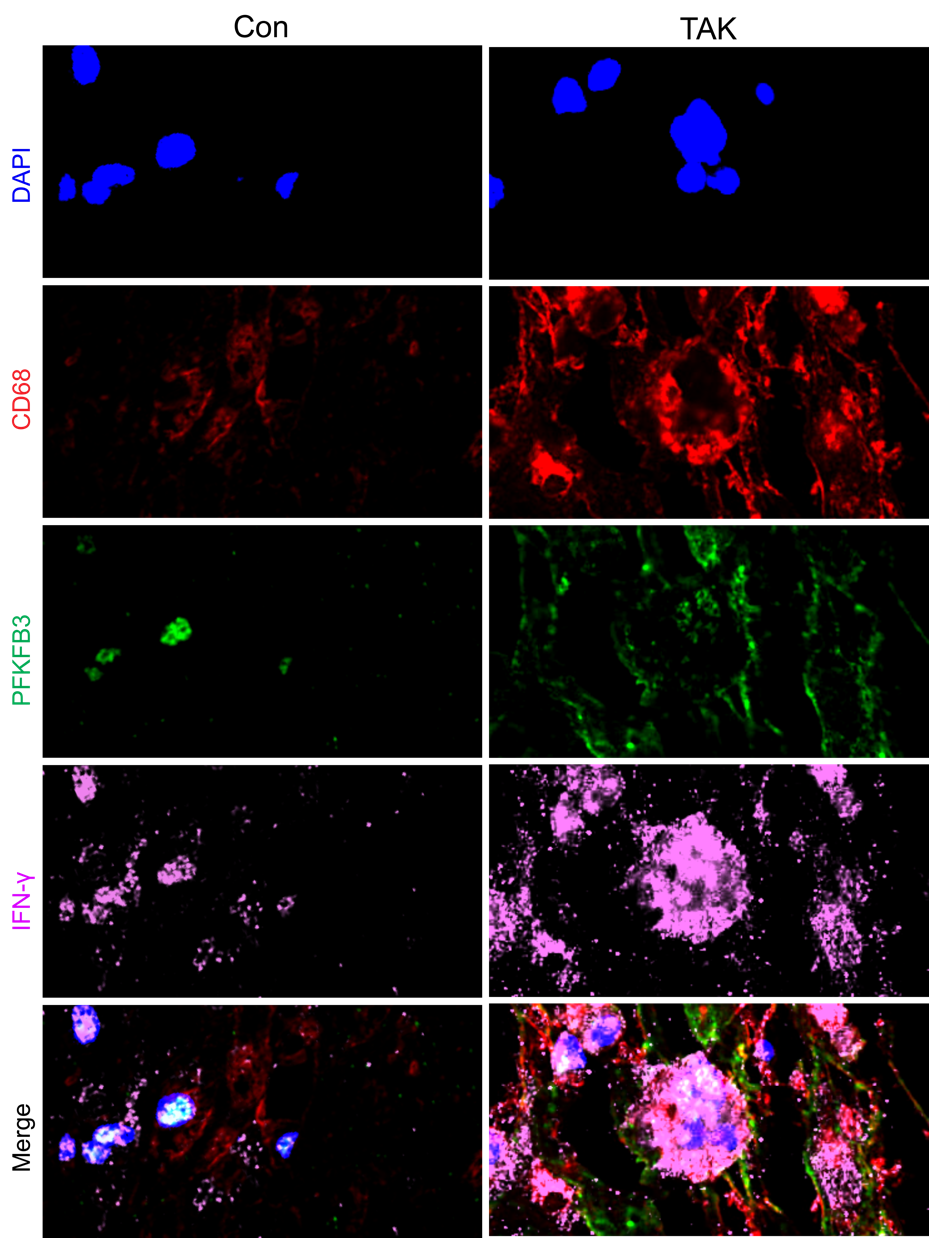


**Supplementary Figure 3.** Validation of Macrophage subtype after polarization.

(A) The representative figure of M0, M1, and M2 in the flow cytometry; (B-E) the expression of macrophage surface markers CD80, HLA-DR, CD163, and CD206; n=6. The culture medium was supplemented with M-CSF (40ng/ml), and LPS (50ng/ml) with IFN-γ (20ng/ml) for inducing M1 differentiation, and IL-4 (20ng/ml) with IL-13 (20ng/ml) for inducing M2 differentiation. Data revealed as *mean± SEM*, *p<0.05.


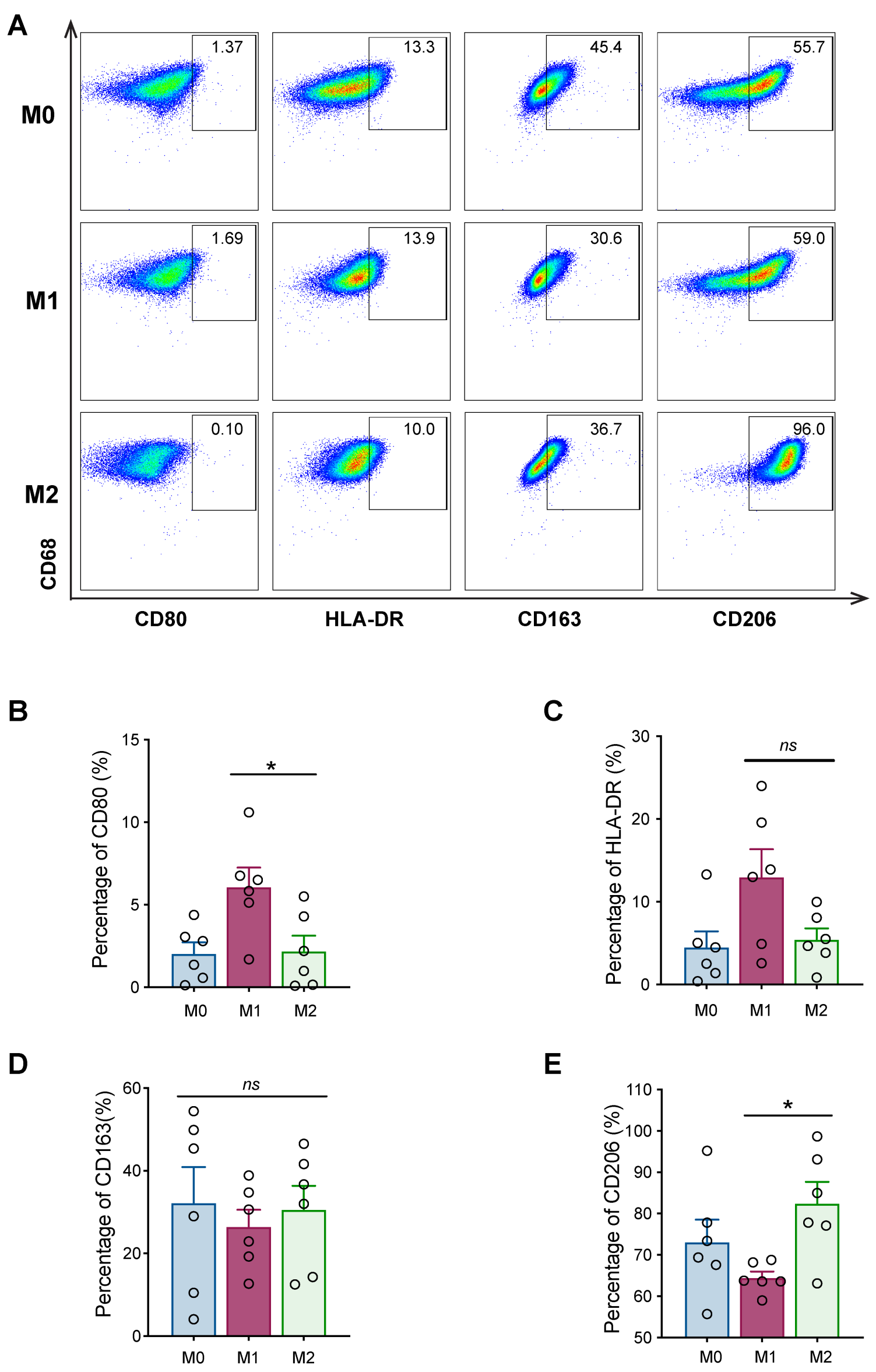


**Supplementary Figure 4.** The expression of metabolism, immune, and cytokines related gene in the presence of PFK-015 (10 μM). n=9. All the culture medium was supplemented with M-CSF (40ng/ml) and LPS (50ng/ml) in inducing M1 polarization stage. The concentration of IFN-γ was 20ng/ml. Data revealed as *mean± SEM*, *p<0.05; ***p<0.001; ****p<0.0001; #p<0.05; ##p<0.01; ####p<0.0001.


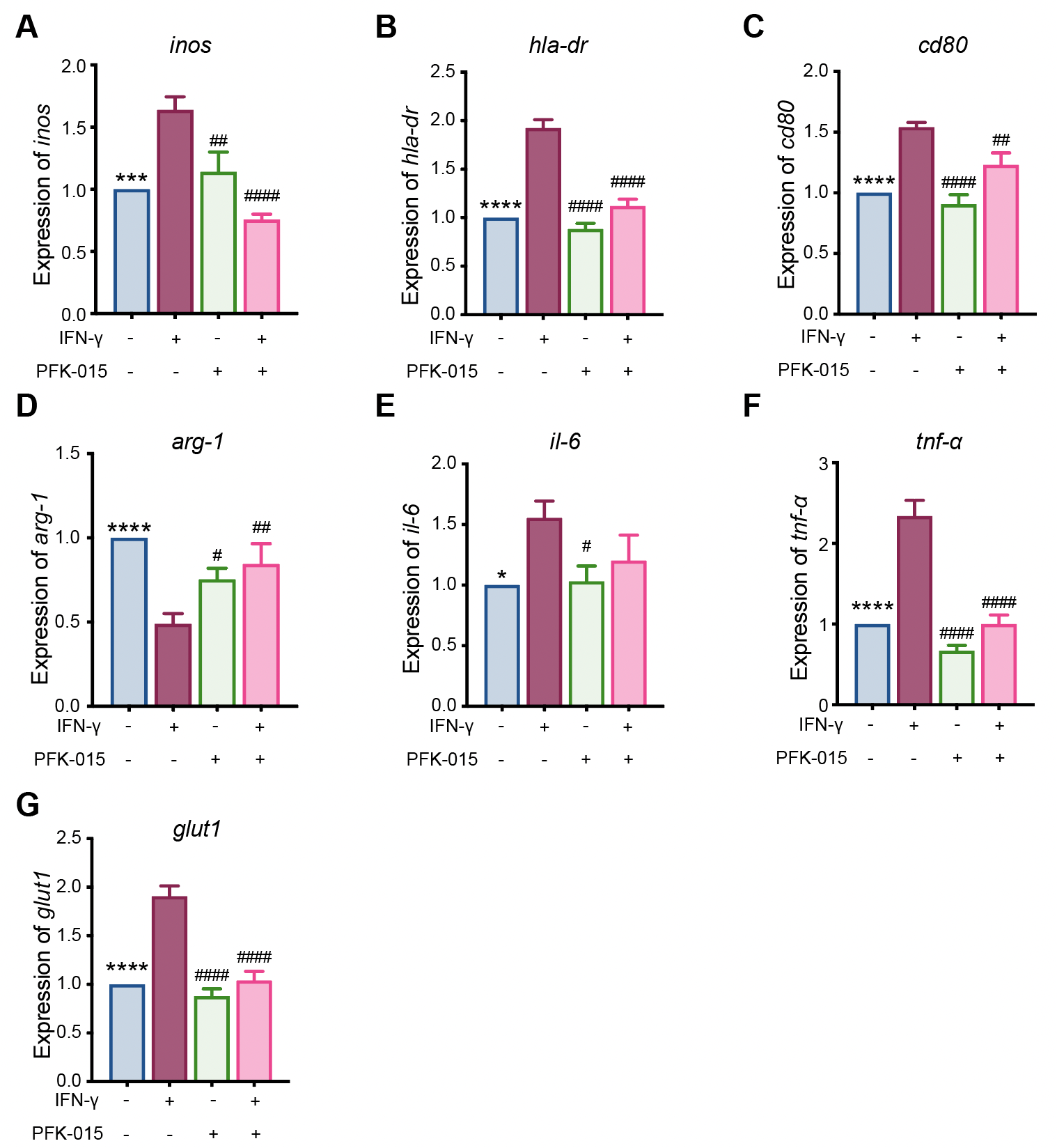


**Supplementary Figure 5**. The influence of PFKFB3 inhibitor PFK-015 on macrophage activity by CCK8.

(A) the effect of different concentration of PFK-015 on macrophage activity at 48 hours; (B) the effect of PFK-015 at 10μM after different stimulus period. All the culture medium was supplemented with M-CSF (40ng/ml). Data revealed as *mean± SEM*, ***p<0.001; ****p<0.0001.


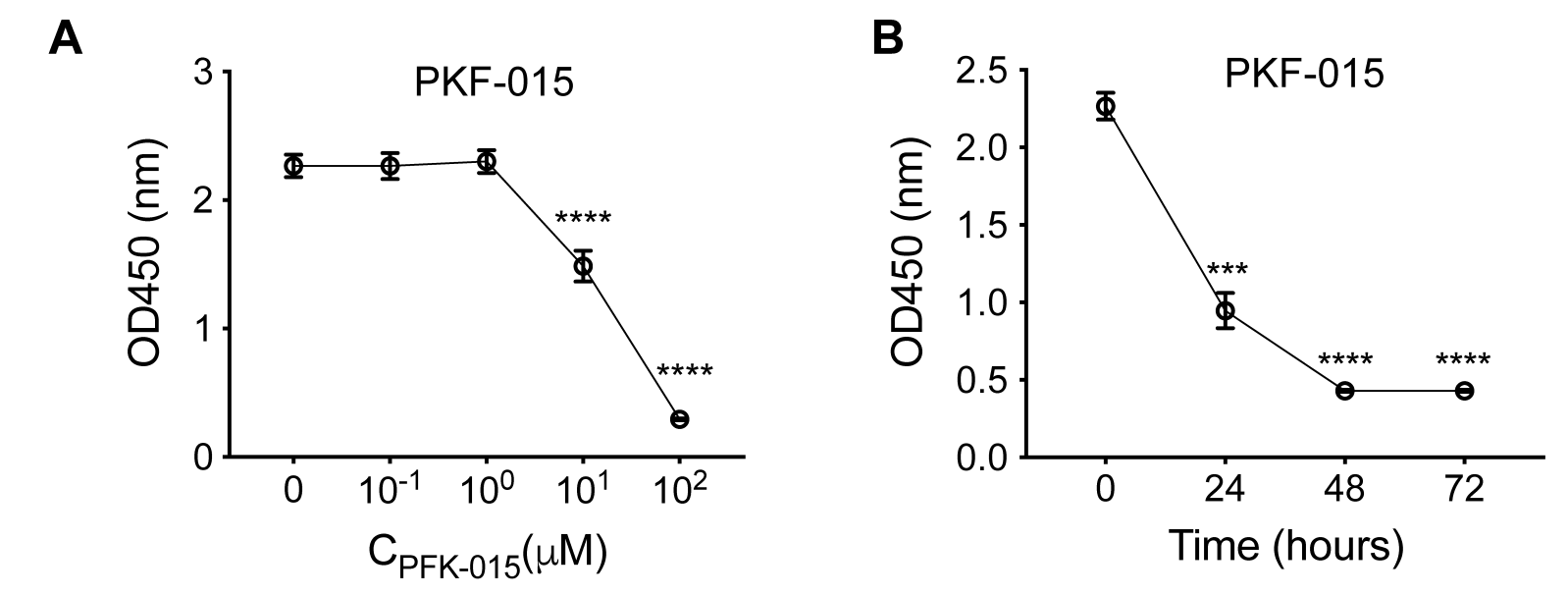


**Supplementary Figure 6.** The isotype control of flow cytometry for macrophage phenotype detection


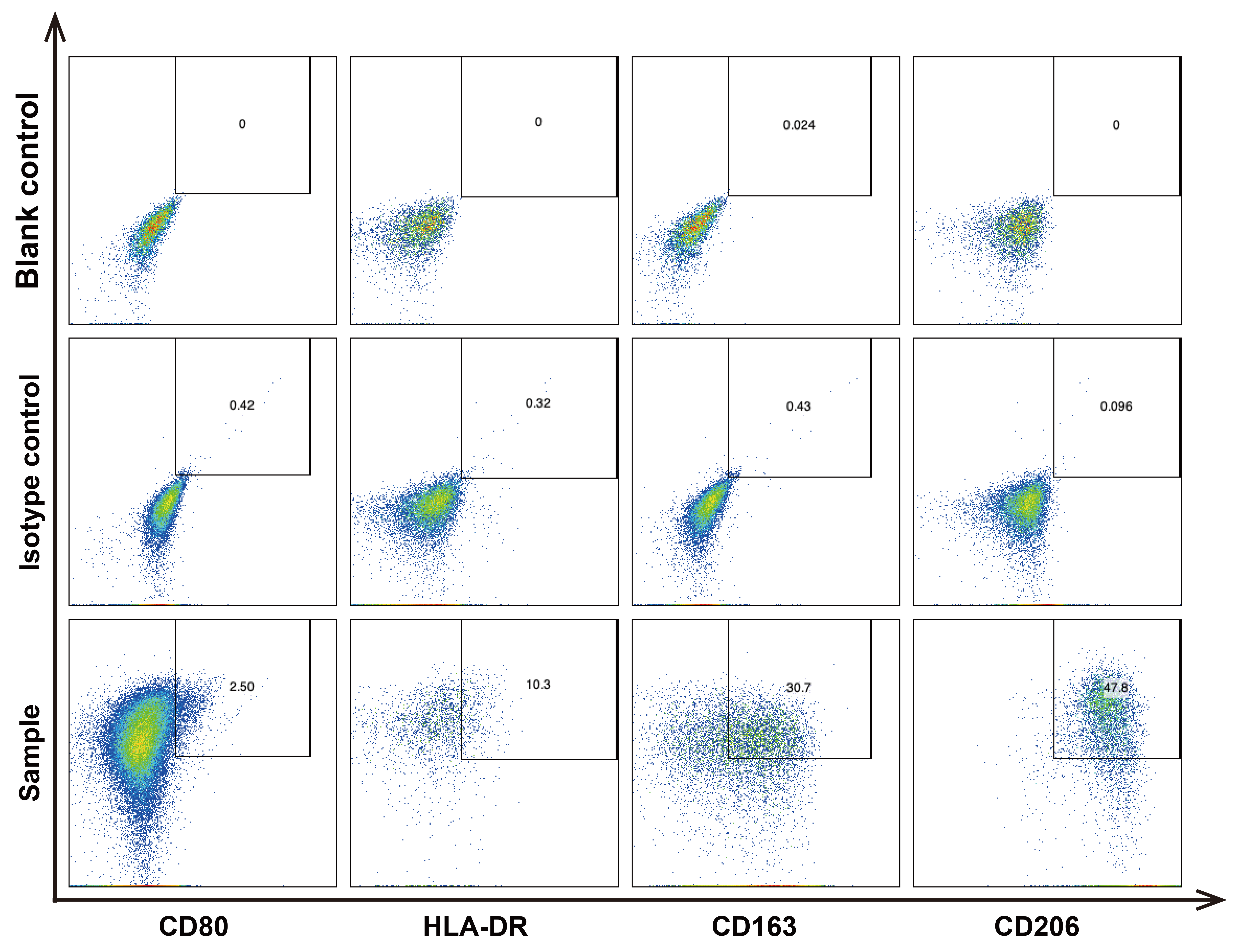


**Supplementary Figure 7.** The phosphorylation of JAK2 in the presence of IFN-γ.

(A-C) The activation of JAK family and STAT1 in the presence of IFN-γ; (D) the phosphorylation of JAK2 under the stimulus of IFN-γ, JAK2 inhibitor (tofacitinib), or STAT1 inhibitor (fludarabine). All the culture medium was supplemented with M-CSF (40ng/ml) and LPS (50ng/ml) in inducing M1 polarization stage. The concentration of IFN-γ was 20ng/ml. Data revealed as *mean± SEM*, *p<0.05; **p<0.01; ***p<0.001.


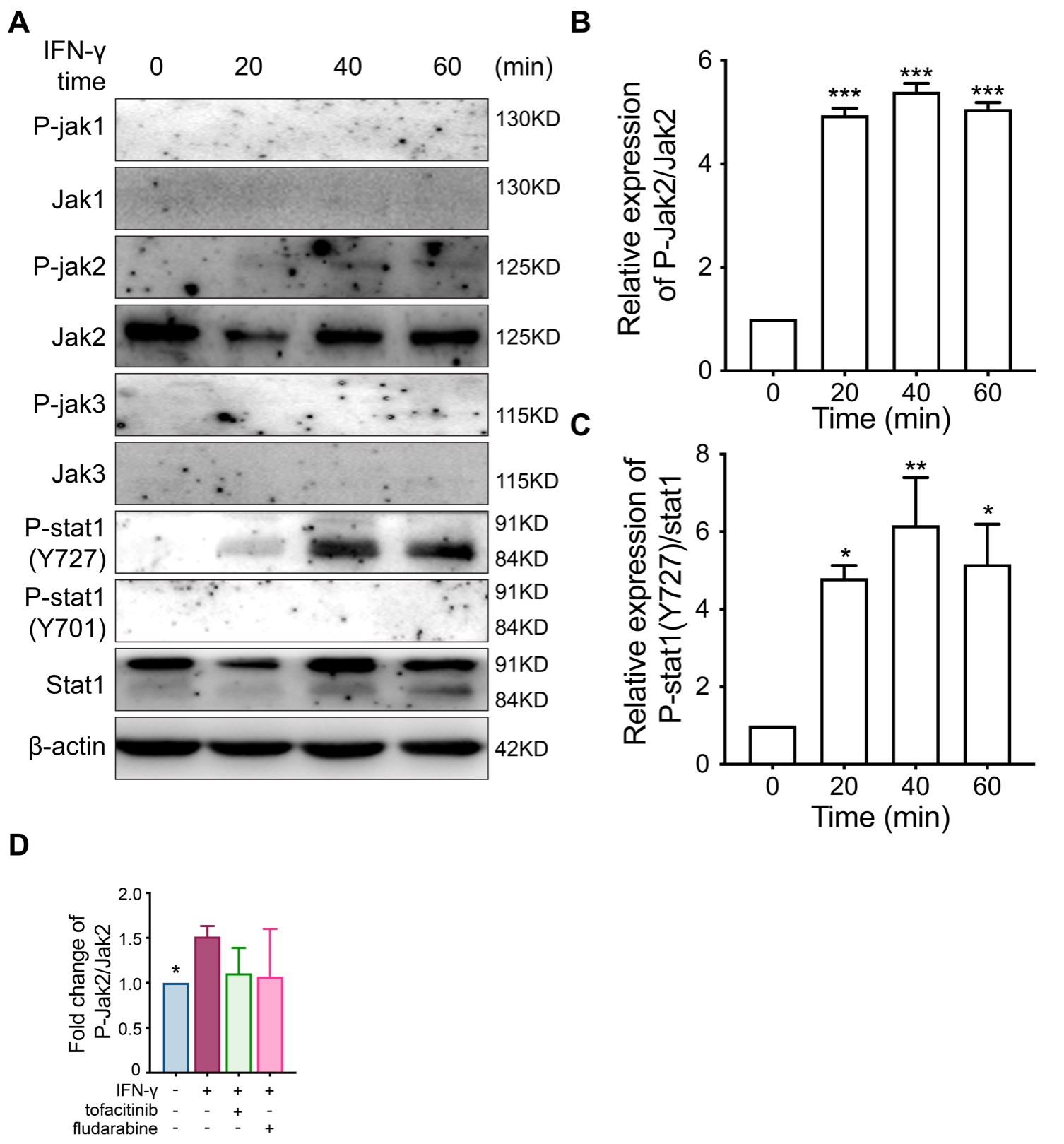


**Supplementary Figure 8.** The glucose metabolism profile in the presence of IFN-γ and signal pathway inhibitors. (A) the ECAR of M1 macrophages under the stimulus of IFN-γ and the JAK inhibitor (tofacitinib, 10nM) and STAT1 inhibitor (Fludarabine, 1nM); (B) the OCR of M1 macrophages under the stimulus of IFN-γ and the JAK inhibitor (tofacitinib, 10nM) and STAT1 inhibitor (Fludarabine, 1nM); (C) the lactate acid in the cell culture supernatant in M1 macrophages under the stimulus of IFN-γ, tofacitinib, and fludarabine. All the culture medium was supplemented with M-CSF (40ng/ml) and LPS (50ng/ml) in inducing M1 polarization stage. The concentration of IFN-γ was 20ng/ml. Data revealed as *mean±SEM*, *p<0.05; **p<0.01; ***p<0.001; ****p<0.0001; #p<0.05; ##p<0.01; ###p<0.001; ####p<0.0001.

**
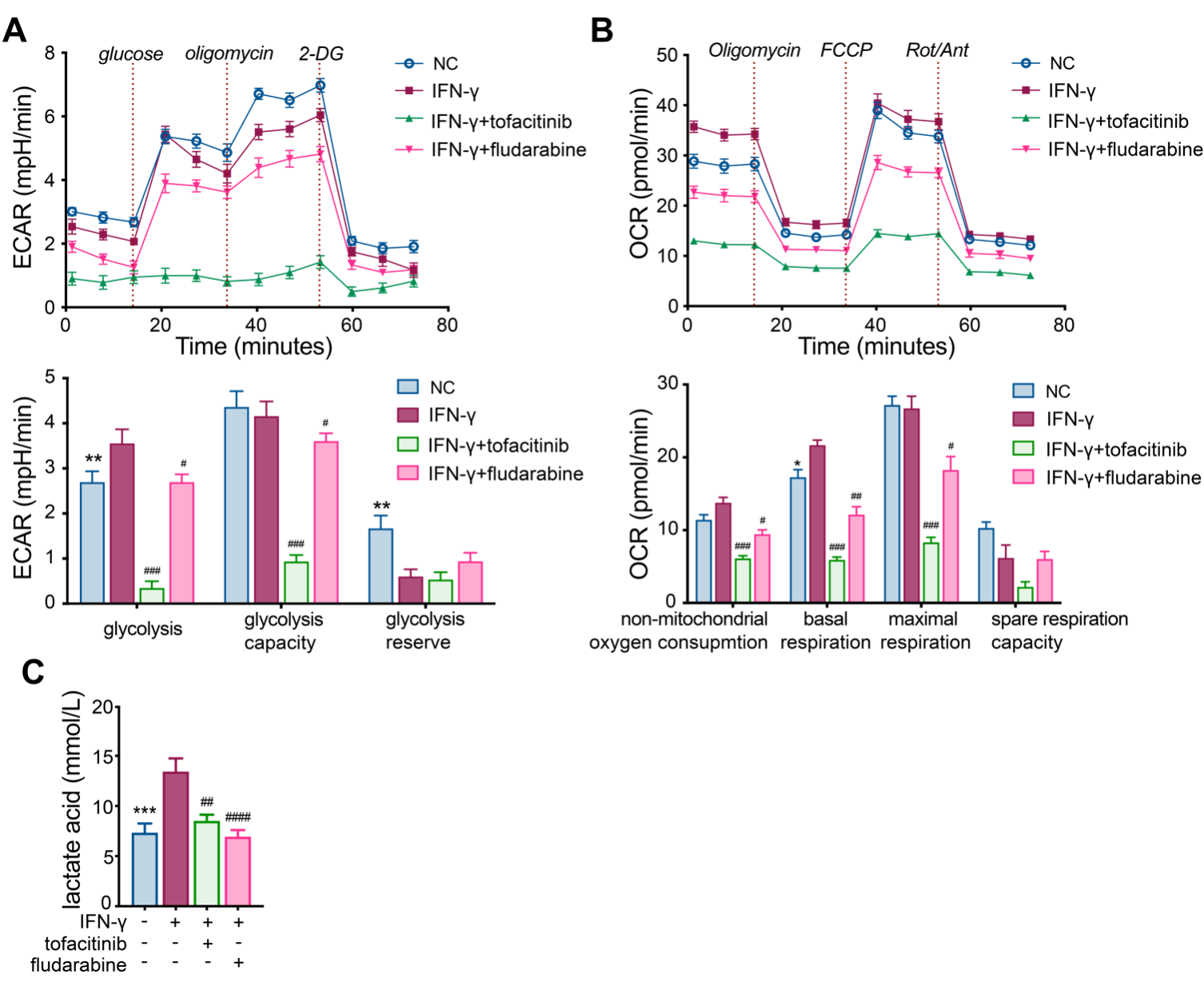
**

**Supplementary figure 9.** The effect of baricitinib on IFN-γ mediated M1 polarization. (A-E) The expression of M1 and M2 phenotype markers in the presence of baricitinib. The culture medium was supplemented with M-CSF (40ng/ml) and LPS (50ng/ml) in the M1 polarization induction stage. The concentration of baricitinib was 50ng/ml. (n=7, * control vs IFN-γ; # IFN-γ vs IFN-γ + baricitinib; data expressed as mean ± SEM and compared by one-way ANOVA; *p< 0.05; # p<0.05; ## p<0.01)


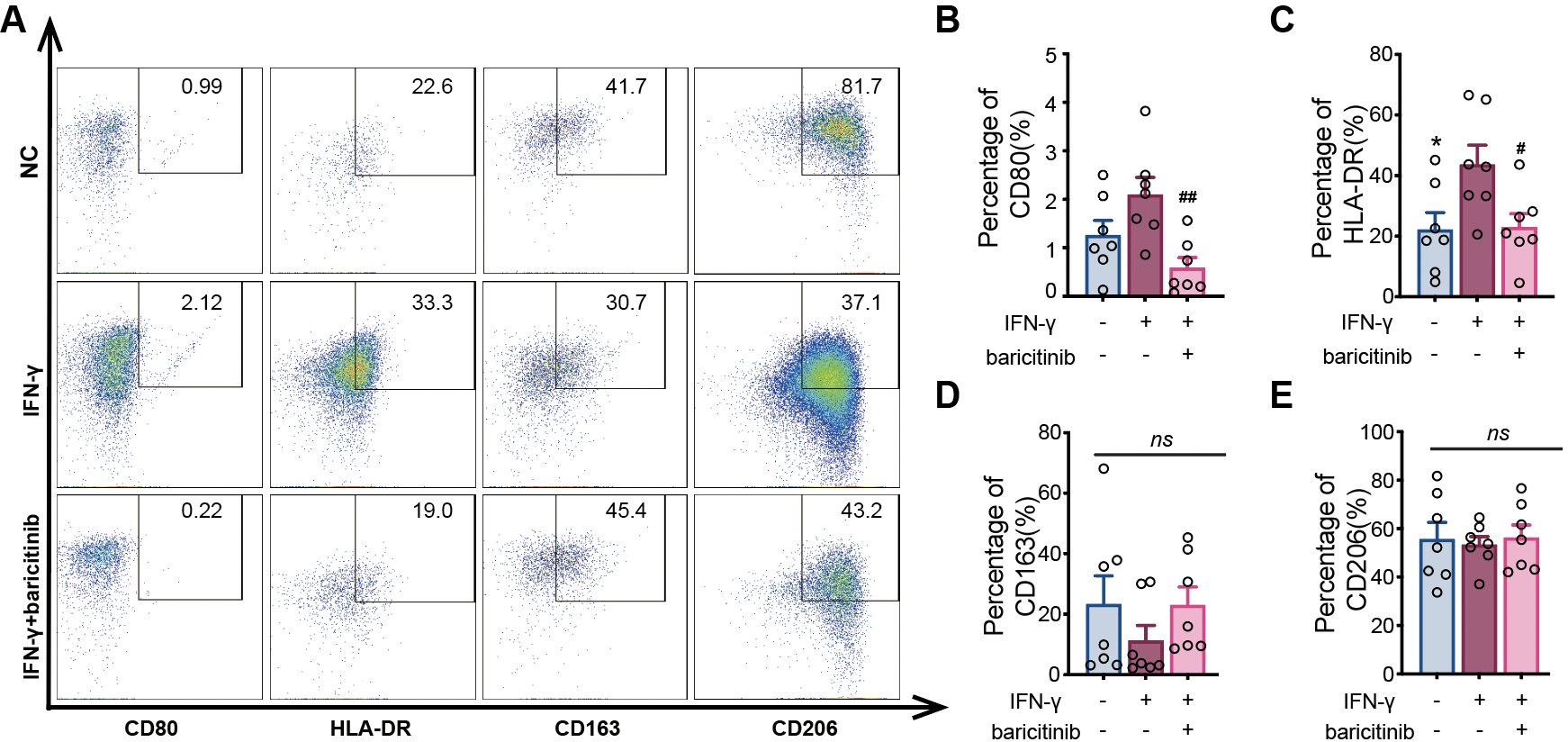


**Supplementary Table 3**. The clinical information of patients used in the baricitinib intervention experiment.

| No. | Age | Sex | Naive | Imaging Type | Active disease | ESR | CRP | IL-6 | Treatment |
| --- | --- | --- | --- | --- | --- | --- | --- | --- | --- |
| 1 | 26 | F | 0 | V | 0 | 13 | 0.3 | 6.2 | prednisone +Methotrexate+ anti-IL-17mAb |
| 2 | 36 | F | 0 | I | 0 | 5 | 1.3 | 2.5 | prednisone +methotrexate+tofacitinib |
| 3 | 54 | F | 0 | V | 0 | 15 | 1.9 | 5.8 | methotrexate +hydroxychloroqunine |
| 4 | 31 | F | 0 | I | 0 | 6 | 1.5 | 3 | Prednisone +Methotrexate+ anti-TNF-alphamAb |
| 5 | 21 | F | 0 | I | 0 | 2 | 0.3 | 3.5 | Prednisone +Methotrexate+ anti-IL-6mAb |
| 6 | 30 | F | 0 | IIb | 0 | 29 | 5.7 | 3.8 | prednisone +leflunomide+tofacitinib |
| 7* | 28 | F | 0 | I | 0 | 101 | 46.2 | 22.4 | prednisone +leflunomide+hydroxycholoroqunine |

Note:

1. F, female;

2. * The 7^th^ patient got the respiratory infection when her blood was obtained.

**Supplementary Figure 10.** The expression of metabolism, immune, and cytokines-related gene in the presence of JAK or STAT1 inhibitor. n=9. All the culture medium was supplemented with M-CSF (40ng/ml) and LPS (50ng/ml) in inducing M1 polarization stage. The concentration of IFN-γ was 20ng/ml. Data revealed as *mean± SEM*, **p<0.01; ****p<0.0001; #p<0.05; ##p<0.01; ###p<0.001; ####p<0.0001.


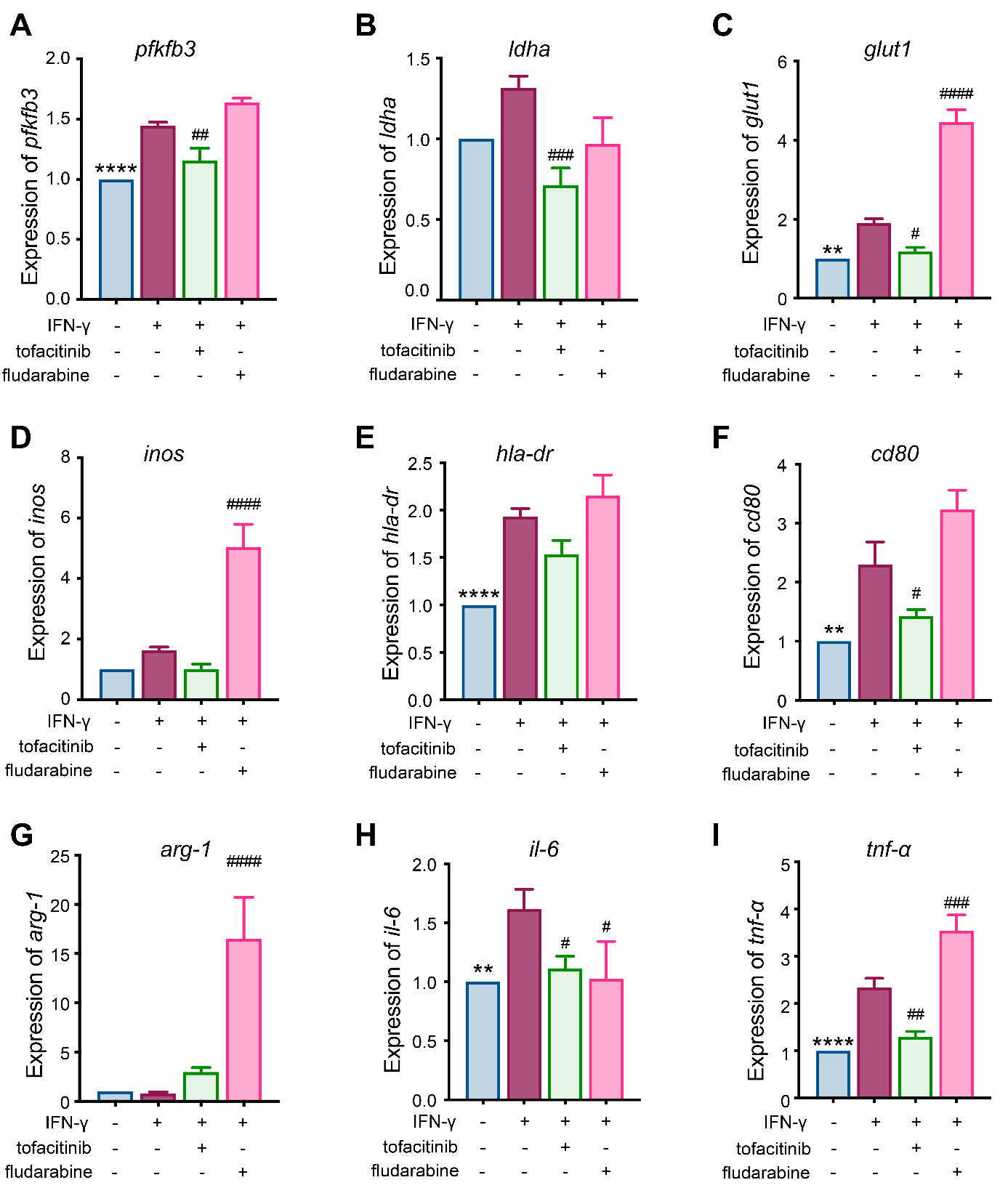

Supplement: Supplementary file 1 — Additional file 1. [file 13075_2022_2960_MOESM1_ESM.docx]
